# Micromanagement of *Drosophila* Post-Embryonic Development by Hox Genes

**DOI:** 10.3390/jdb10010013

**Published:** 2022-02-18

**Authors:** Alexandra D. Buffry, Alistair P. McGregor

**Affiliations:** Department of Biological and Medical Sciences, Oxford Brookes University, Gipsy Lane, Oxford OX3 0BP, UK; a.buffry@brookes.ac.uk

**Keywords:** Hox genes, *Drosophila*, post-embryonic development, gene expression

## Abstract

Hox genes function early in development to determine regional identity in animals. Consequently, the loss or gain of Hox gene expression can change this identity and cause homeotic transformations. Over 20 years ago, it was observed that the role of Hox genes in patterning animal body plans involves the fine-scale regulation of cell fate and identity during development, playing the role of ‘micromanagers’ as proposed by Michael Akam in key perspective papers. Therefore, as well as specifying where structures develop on animal bodies, Hox genes can help to precisely sculpt their morphology. Here, we review work that has provided important insights about the roles of Hox genes in influencing cell fate during post-embryonic development in *Drosophila* to regulate fine-scale patterning and morphology. We also explore how this is achieved through the regulation of Hox genes, specific co-factors and their complex regulation of hundreds of target genes. We argue that further investigating the regulation and roles of Hox genes in *Drosophila* post-embryonic development has great potential for understanding gene regulation, cell fate and phenotypic differentiation more generally.

## 1. Introduction

Hox genes encode a family of conserved transcription factors (TFs) that pattern the antero–posterior (A–P) body axis of all animals [1,2,3,4]. The Hox genes are arranged in clusters in most animals, and their spatial expression along the A–P axis reflects their physical position along the DNA of the cluster, a phenomenon referred to as spatial collinearity [5,6,7,8].

**Figure 1:**
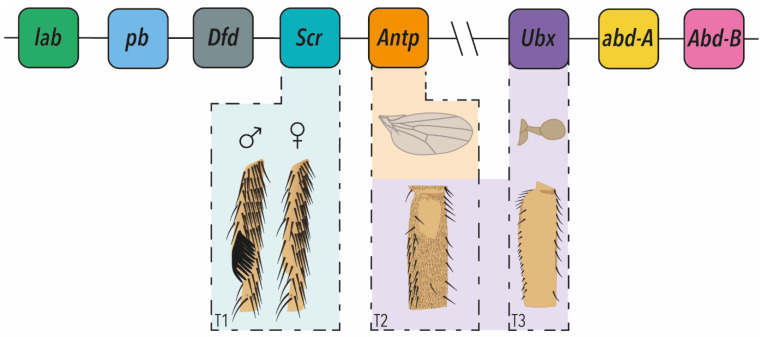
The Hox clusters of *Drosophila melanogaster* and micromanagement of appendage morphology. The Antennapedia and Bithorax clusters are shown above drawings of T1, T2 and T3 appendages whose morphology is managed by the Hox genes indicated. Males exhibit modified bristles called sex combs on T1, which is regulated by *Scr*. T2 wing morphology is regulated by *Antp*. T2 legs exhibit a *Ubx*-dependent variably sized trichome-free patch of cuticle on the proximal posterior femurs. On T3, the hind-wings have evolved into halteres, which are specialised balancing organs. This is achieved through *Ubx* repression of wing development, and promotion and fine-scale sculpting of haltere development. *Ubx* also modulates the morphology of the T2 and T3 legs.

Much of the pioneering work on Hox genes that catalysed a massive expansion in evolutionary developmental biology research was carried out by studying these genes in the fruit fly, *Drosophila melanogaster* [9,10]. *Drosophila* has eight Hox genes split between the Antennapedia (*labial*, *proboscipedia*, *Deformed*, *Sex-combs reduced* (*Scr*) and *Antennapedia* (*Antp*)), and Bithorax (*Ultrabithorax* (*Ubx*), *abdominal-A* (*abd-A*) and *Abdominal-B* (*Abd-B*)) complexes (Figure 1). Note that most animals have a full set of ten Hox genes but in *Drosophila* Hox3 (*bicoid* and *zerknüllt*) and *fushi tarazu* no longer have Hox functions [5,7,8,11] (Figure 1).

It was considered that Hox genes dictate the identity of individual segments during embryogenesis, with posterior Hox genes exhibiting prevalence over anterior Hox genes [5,6,7,8]. This classical view of Hox gene function reflected gross changes in segmental identify observed when the expression of a Hox gene was lost—homeotic transformations [2,12,13]. For example, the loss of *Ubx* expression in the third thoracic segment (T3) results in transformation of this segment into an additional second thoracic segment (T2) and a fly with two pairs of wings, instead of the usual halteres on T3 [2]. In this model, Hox genes can be thought of as acting like switches in each segment, with the identity of all cells in a segment being selected by the most posterior Hox gene expressed in that segment [11,13]. This decision is then ‘locked-in’ by the expression of trithorax group or polycomb group of chromatin modifiers to maintain or repress Hox expression in the segment.

However, this strict selector (or master) model of Hox gene function was challenged from an evolutionary perspective, as well as by emerging new insights into Hox function in the 1990s. In seminal perspective papers in 1998, Michael Akam elegantly argued that the selector model was an over-simplification and in fact, Hox gene function is actually far more nuanced [11,13]. He likened Hox genes to ‘micromanagers’ that can regulate cell fate decisions and fine-scale morphology, not only within ‘their’ segments, but also in other segments during development (Figure 1) [11,13]. One important implication of this is that changes in Hox gene expression, interactions and target genes contribute to the evolution of the fate of specific cells within segments and changes in fine-scale morphology.

Studies of postembryonic development in *Drosophila* have subsequently shown that the micromanager model does more accurately reflect the functionality of Hox TFs, and their role in morphological evolution. These studies have shown that the spatial and temporal expression of Hox TFs, as well as their relative concentration among cells in the same segment, rather than simply on or off, is required for the correct fine-scale development of segments and sculpting of adult tissues (Figure 1). This is achieved through the regulation of Hox gene expression and function by co-factors, to activate or repress potentially hundreds of target genes, to precisely control cell fate. A growing number of studies also evidence how differences in Hox expression and interactions underlie fine-scale evolution including appendage morphology and pigmentation (e.g., [14,15,16]).

In this article, we review key studies about the regulation and function of Hox genes during postembryonic development in *Drosophila* that evidence their roles as micromanagers. Furthermore, we argue that further understanding these roles of Hox genes and how they are wired into post-embryonic gene regulatory networks can continue to provide key new insights into gene regulation, cell fate and phenotypic differentiation more generally.

## 2. Hox Gene Regulation of Fine-Scale Phenotypes in *Drosophila*

### 2.1. Wings (T2) versus Halteres (T3)

The second thoracic segment (T2) of *Drosophila* develops wings, whereas the third thoracic segment (T3) develops halteres, which are specialised organs evolved from hind-wings that aid balance (Figure 1). *Ubx* was previously thought to be the sole Hox gene responsible for the differential development between T2 and T3 [17]. Indeed, in T3 *Ubx* suppresses wing development, while it was previously thought that wing development in T2 was ‘Hox free’ [18,19]. Recently, however, Paul and colleagues (2021) showed that *Antp* is actually expressed in the wing pouch from the first (L1) to third (L3) larval instars [20]. This early expression of *Antp* was previously missed, with expression only seen later in the hinge and notum [18]. This more recent work revealed an early role for *Antp* in defining the wing margin, suggesting that it is not just suppression of *Ubx* that underlies the development of wings. Furthermore, this study found evidence that the correct dose of Hox gene expression is critical for the correct size and shape of structures, because too much or too little *Antp* results in smaller wings [20].

The idea that Hox dosage may be more important than the Hox gene expressed during the development of the haltere was observed previously by Casaras et al. (1996) [21]. For example, when ectopically expressed, Ubx, Abd-A and to some extent Abd-B, can transform wing tissue into haltere tissue. In fact, they showed that Abd-A can fully, and Abd-B partially substitute for Ubx in haltere development [21]. More recent observations from Paul and colleagues (2021) showed that Antp and Abd-A can actually rescue T3 halteres from wing development in the absence of the wing suppressor Ubx [20]. It appears that a high Hox dose is required in halteres and a lower dose in wings, but curiously whether this is for *Antp*, *abd-A* or *Ubx* in both primordia may not be important, presumably because the activity of the Hox TF is modulated by co-factors in each segment and generic binding site recognition means they may be able to regulate the same target genes. The authors also suggest that such differences in Hox dosage could explain the evolution of differences in size and shape between T2 and T3 wing appendages more broadly across insects, and not just *Drosophila* [20].

*Ubx* not only suppresses wings in T3, but actively promotes haltere development and morphology by directly regulating genes involved in extracellular matrix dynamics [17,22,23]. Indeed, it has been shown that *Ubx* interacts with many of the genes located in the wing patterning network to repress the specification of wing-specific morphology, this leads to the balloon shape structure of the haltere, and a lack of wing veins and bristles [17,23,24,25]. Additionally, *Ubx* has been shown to affect the fine-scale patterning of the haltere, including the morphology of the campaniform sensilla [17]. *Ubx* has also been implicated in the control of haltere size through interaction with the Dpp signalling pathway [26,27]. It has been shown that decreasing the dosage of *Ubx* in the halteres increases the size of these structure, and vice versa [28]. Recent work by Delker and colleagues (2019) investigated this further and showed that differential activity of *Ubx* within the haltere primordia is required for their correct morphology [29]. The authors demonstrated that although *Ubx* creates a binary switch to control wing versus haltere identity, the levels of *Ubx* actually differ within the compartments of the haltere along the proximo–distal axis. The distal compartment of the haltere has higher expression of *Ubx* than the proximal compartment, which is achieved by a negative autoregulatory loop through the known *Ubx* enhancer *abx*. The *abx* enhancer was originally identified as a cis-regulatory module required for the activation of *Ubx* in the haltere [30,31]. However, this more recent work from Delker and colleagues (2019) shows the same cis-regulatory module is needed for both activation and repression, a process that is achieved by small clusters of low-affinity TF binding sites that are bound by Ubx and its cofactors, Extradenticle (Exd) and Homothorax (Hth) [29]. These sites are critical to achieve proximo–distal expression bias in the haltere, which is crucial for correct morphology.

In a follow up study, Loker and colleagues (2021) used ATAC-seq and binding assays to further investigate the ability of Ubx to act as a repressor and/or activator in a cell type-specific manner [32]. In the distal hinge of the haltere (high Ubx expression), the Ubx-Hth-Exd motif is enriched in accessible chromatin, suggesting that in this context Ubx utilises its cofactors for gene activation [32]. Conversely, in the same compartment of the haltere, in cells where the chromatin accessibility is lower and there is no Hth, Ubx binds as a monomer to repress gene expression [32]. Interestingly, in proximal haltere cells (low Ubx expression) there is also enrichment for Ubx-Hth-Exd motifs in accessible chromatin, suggesting that, in this particular context, Ubx works with these cofactors to achieve a repressive role. It seems that the pouch of the haltere, is free from Hth expression and neither Hth or Exd are required for Ubx-dependent development in this region [33,34]. Further analysis of a regulatory element that is repressed by Ubx in the pouch region shows a tandem array of Ubx binding sites, which could suggest that Ubx multimerisation might negate the need for cofactor binding [35]. Altogether, this evidence suggests that transcriptional regulation by Ubx is dependent on both the position along the proximo–distal axis and the availability of co-factors [32]. This study also suggests that Ubx can play a role in altering chromatin accessibility in a cell type-specific manner, as has also been shown for some mammalian Hox genes [36,37,38].

### 2.2. Leg Morphology

The three serially homologous leg pairs of *Drosophila* differ in their size, shape and finer-scale morphology [39]. These differences are regulated, in part, by Hox genes acting at different levels in the gene regulatory networks that specify these appendages and their appearance.

T1 legs display bristle patterns that differ from the T2 and T3 legs [39,40,41,42]. The ventral-anterior of the distal part of the T1 tibia and posterior part of the tarsus have transverse rows of bristles, rather than longitudinal rows found on other leg surfaces and T2 legs (Figure 1) [39,43]. In males, the distal transverse bristle row on the first tarsal segment is rotated by 90 degrees, to form a sex comb made up of modified bristles [14,39,44] (Figure 1). The sex combs are fast-evolving, secondary sexual structures that the male uses to grasp the female during copulation [14,44,45].

It has been shown that these T1 and male-specific features are regulated by precise spatial and temporal expression of *Scr* [46]. This Hox gene is expressed throughout T1, but it has higher expression in the region where transverse bristle rows will later form in both sexes [42,43]. *Scr* expression is further elevated at the pupal stage in the first tarsal segment of males, corresponding to where the sex comb will develop; however, this expression is subsequently downregulated in the bristle precursor cells [42,43,47].

Eksi and colleagues (2018) identified enhancers that drive these aspects of *Scr* expression in T1 legs and demonstrated that removal of the upstream enhancer results in the loss of both the transverse bristle rows in both sexes, and the sex combs in males, consistent with the requirement for *Scr* to generate these bristle patterns [42,43]. This work shows that *Scr* not only specifies T1 identify, but is integrated into a postembryonic gene regulatory network to micromanage the morphology of T1 legs. *Scr* expression in T1 is directly activated by Distal-less (Dll) and repressed by Engrailed (posteriorly) and Bric-a-brac (distally), and in turn Scr regulates Doublesex and Delta [42,43,48,49]. Therefore, *Scr* links the leg patterning gene network to the sensory organ specification pathway, in both males and females, as well as regulating the sex determination pathway to generate male-specific sex combs (Figure 1) [42,43,48,49].

*Ubx* helps to determine differences in the size and shape of T2 and T3 legs [50]. However, this Hox gene also regulates the fine-scale morphology of these appendages [16,50,51]. Cells on the distal part of the T2 femur project trichomes (non-sensory actin protrusions), whereas more proximal cells are free from trichomes, and the cuticle is smooth, forming the so-called ‘naked valley’ [16,50,51,52,53] (Figure 1). It has been shown that a proximo–distal gradient of *Ubx* expression in the pupal femur results in naked valley formation through repression of the proximal trichomes [16]. Furthermore, higher expression of *Ubx* in the femur increases the size of the naked valley, as observed in *D. simulans* [16] (Figure 1). *Ubx* expression in pupal T2 legs is regulated by a recently discovered enhancer in the 3rd intron [54]. Curiously, knockdown of *Dll* in T2 pupal legs expands the naked valley and therefore it is possible that Dll, at least in directly, represses *Ubx*, in contrast to the role of *Dll* in activating *Scr* in T1 [42,54]. Although the direct target genes of *Ubx* in T2 femur cells have yet to be identified, it acts via *microRNA-92a*, which blocks translation of target genes of the trichome activator, Shavenbaby and underlies natural variation in naked valley size [52,53,54,55].

T3 legs exhibit transverse bristle rows on the posterior of the distal tibia, basitarsus and second tarsal segments [39,43]. While *Ubx* is expressed broadly in T3 pupal legs, it is locally upregulated on posterior T3 pupal leg segments, where it directs formation of transverse bristle rows via repression of *Delta*, for example on the T3 basitarsus [43]. This role of *Ubx* is similar to that played by *Scr* in T1 legs [43].

### 2.3. Micromanagement of Other Aspects of Post-Embryonic Development

The above examples highlight the in-depth analysis that has been carried out to understand *Scr*, *Antp* and *Ubx* regulation and function in postembryonic patterning of the thoracic appendages (Figure 1). However, it is clear that Hox genes also play roles in managing different aspects of the development of other segments during postembryonic development.

Singh and Mishra (2014) showed that, as well as specifying segments A2 to A6 through suppression of *Ubx*, *abd-A* manages various aspects of the formation of the adult epithelia by promoting the proliferation of histoblast nest cells, and activating apoptosis of larval epithelial cells [56]. This shows that *abd-A* is wired into different gene regulatory networks that combine to specify the overall development and identify, and perhaps even the size of segments A2 to A6 [56]. This work also highlighted the importance in the dosage of Abd-A and Abd-B in determining segment morphology and challenged the ubiquity of posterior prevalence rule because these two TFs are expressed in the same nuclei in A5 and A6, but perform distinct functions, presumably through the regulation of different batteries of target genes [56]. *Abd-B* also plays other roles in patterning the most posterior abdominal segments, including the specification of the posterior spiracles. In this role *abd-B* acts through dynamic feed-forward and feedback loops with JAK/STAT signalling to manage posterior spiracle organogenesis from embryogenesis through to postembryonic development [57].

The *Drosophila* larval oenocytes are specialised cells that form in clusters beneath the developing epidermis, and play roles in the synthesis and metabolism of lipids and hydrocarbons (reviewed in [58]). It had previously been shown that binding of Abd-A to a *rhomboid (rho)* cis-regulatory module is required for the formation of larval oenocytes [59,60], this process was further investigated by Li-Kroeger and colleagues (2012) who discovered that complex interplay between Abd-A and its co-factors is required for the formation of these specialised cells [61]. In this context, it appears that Abd-A forms an activating complex composed of Hth, Exd and Pax2. The authors suggest that Abd-A uses the same binding site in the *rho* cis-regulatory module to induce gene activation in a cell-specific manner by interacting with Exd/Hth to restrict binding of other TFs, and also by forming an activating complex with Pax2 [61]. This cooperation between Abd-A and Pax2 is thought to also regulate additional target genes; however, further testing is required to elucidate the role this complex may play during development [61].

*abd-A* and *Ubx* are also required during metamorphosis for the development of the adult *Drosophila* heart [62]. In this context, regulation of the expression of these Hox genes and the modulation of Abd-A activity by ecdysone are required for the remodelling cardiac tube along the antero–posterior axis [62].

Hox genes are also involved in specifying the identity of the nervous system along the antero–posterior axis. However, we are beginning to understand in detail how they fine-tune the fate and function of neurons during postembryonic development to generate the adult nervous system, for example the morphology of motor neurons and neuromuscular networks as recently reviewed by Joshi and colleagues [63].

Finally, it has been shown that Abd-B regulates the male-specific pigmentation of male *Drosophila* through the regulation of *yellow* [15]. Changes in this interaction have been shown to underlie the loss of pigmentation in at least one lineage, providing an excellent example of how changes in Hox function during postembryonic development can contribute to phenotypic evolution [15].

## 3. Hox Target Genes in Post-Embryonic Development

To fully understand the roles of Hox genes in developing tissues, it is crucial to identify the direct targets of these TFs across developmental stages and among the different cells of the tissue. To date, only a few studies have attempted to identify the genome wide direct targets of Hox TFs during postembryonic development in *Drosophila,* especially after puparium formation [64,65,66,67]. This work has shown that Hox regulation of downstream gene expression is complex and supports the view that these TFs micromanage the development and morphology of tissues.

To identify direct targets of Ubx underlying the transformation of wings to halteres, Pavlopoulos and Akam (2011) induced over-expression of this TF at different stages of wing development to repress the specification of wings and promote haltere fate [67]. Assaying changes in gene expression compared to controls allowed them to infer the primary targets of Ubx as opposed to secondary/indirect changes in gene expression. They found that this Hox TF directly regulates the expression of hundreds of target genes. Furthermore, Ubx acts as both an activator and repressor and strongly regulates some target genes, while subtly modulating the expression of others [67]. Consistent with the role of a micromanager, they found that Ubx regulates different sets of target genes at different developmental stages and these targets represent a wide range of genes including other TFs, signalling proteins, adhesion molecules and cuticular and cytoskeletal components [67]. This work on Ubx targets, together with analysis of how *Ubx* itself is regulated and functionally modulated (see above), shows how a Hox gene helps to build and sculpt the fine-scale morphology of an organ during development.

## 4. Future Work on Roles of Hox Genes in Post-Embryonic Gene Regulatory Networks

Further studies of Hox genes in *Drosophila* postembryonic development have great potential to better understand the roles of these genes and how they regulate cell fate to manage the shape, size and fine-scale morphology of segments, organs and appendages. Furthermore, this can continue to provide fundamental insights into the modulation of TF function and the topology and dynamics of gene regulatory networks more generally.

However, much needs to be done to understand how Hox genes are integrated into gene regulatory networks. This requires further detailed characterisation of the regulation of Hox genes, including the identification of the enhancers and direct TF regulators of these genes during different stages of their postembryonic expression. It is clear that Hox gene autoregulation and cofactors, such as Exd and Hth, greatly influence Hox function, and the precise and often opposing roles they play, even between adjacent cell populations. We need to better understand the molecular mechanisms involved in how co-factors and feedforward and feedback loops modulate Hox activity through influencing protein expression and functionality directly, or in target gene selection among cells during segment and organ development [57]. Clearly there is a great need for further studies to identify the direct genome wide targets of all the Hox genes in different tissues and at different developmental stages. Techniques such as Cut&Run now make this more feasible by providing information on Hox binding at high resolution from small amounts of tissue [68,69]. Furthermore, single-cell approaches for RNA-Seq and ATAC-seq, for example, can help identify the targets and differences among cells in a tissue and help to understand how they are regulated [70,71]. Finally, CRISPR/Cas9 editing now allows candidate modulators and targets of Hox genes, enhancers and even individual binding sites to be manipulated, and precise further testing of the roles of Hox genes in specific tissues and cells types in postembryonic contexts [72,73].
